# Decoding the immunoregulatory functions of ALKBH5 in the tumor microenvironment

**DOI:** 10.3389/fimmu.2025.1709260

**Published:** 2026-01-05

**Authors:** Chunhong Li, Xiulin Jiang, Yixiao Yuan, Qiang Wang

**Affiliations:** 1 Department of Oncology, Suining Central Hospital, Suining, Sichuan, China; 2 Department of Systems Biology, City of Hope Comprehensive Cancer Center Biomedical Research Center, Monrovia, CA, United States; 3 Department of Gastrointestinal Surgical Unit, Suining Central Hospital, Suining, Sichuan, China

**Keywords:** ALKBH5, cancer immunotherapy, immune checkpoint, m6A demethylase, tumor microenvironment

## Abstract

N^6^-methyladenosine (m^6^A) modification represents the most prevalent internal RNA modification and plays a pivotal role in regulating RNA metabolism and cellular function. As a major m^6^A demethylase, ALKBH5 not only orchestrates tumor cell proliferation, migration, and metabolic reprogramming but also exerts profound effects on the tumor immune microenvironment (TME). Accumulating evidence has revealed that ALKBH5 regulates immune cell recruitment and function, including CD8^+^ T cells, Tregs, NK cells, and tumor-associated macrophages, by modulating chemokines, cytokines, and metabolic pathways in an m^6^A-dependent or independent manner. Moreover, ALKBH5 influences immune checkpoint expression, such as PD-L1, thereby shaping antitumor immune responses and affecting the efficacy of immunotherapy. Upstream regulatory signals, including hypoxia, inflammation, and epigenetic modifications, further fine-tune ALKBH5 expression and activity. Given its dual roles in promoting or suppressing antitumor immunity depending on tumor type and context, ALKBH5 emerges as both a potential biomarker and therapeutic target. Understanding the multifaceted functions of ALKBH5 in tumor immunity provides new insights into precision immunotherapy and may guide the development of novel combination strategies to overcome resistance.

## 1 Introduction

In recent years, epitranscriptomics has emerged as a critical layer of gene expression regulation and has attracted broad attention. Among the various chemical modifications, m^6^A is recognized as the most prevalent and functionally significant internal modification in eukaryotic mRNAs and diverse classes of non-coding RNAs (1, 2). m^6^A is a dynamic and reversible process: it is deposited by the “writer” complex (such as METTL3–METTL14–WTAP), recognized by “reader” proteins (including the YTH family, IGF2BPs, and HNRNPs) that mediate downstream fate decisions (splicing, nuclear export, translation, and decay), and can be removed by specific “eraser” enzymes, thereby enabling fine-tuned, dynamic regulation of RNA metabolism (3–7). By modulating transcript stability, alternative splicing, translational efficiency, and non-coding RNA function, m^6^A plays crucial roles in development, stem cell fate determination, neural plasticity, metabolic homeostasis, and immune responses (3).

Within the enzymes that catalyze or reverse these modifications, the AlkB homolog (ALKBH) family belongs to the Fe²^+^/α-ketoglutarate–dependent dioxygenase superfamily, comprising ALKBH1–8 in mammals (8, 9). These members have diverse substrates and biological functions: for instance, ALKBH2/3 primarily participate in DNA alkylation damage repair (10), ALKBH8 mediates tRNA modifications linked to translation, whereas FTO and ALKBH5 have been identified as RNA m^6^A demethylases, directly contributing to the reversibility of the epitranscriptome (11–13). ALKBH5 is predominantly localized in the nucleus and specifically removes m^6^A marks from target transcripts, thereby regulating their splicing, nuclear export, stability, and translation (14). Its roles have been reported in embryonic development, stem/tumor stem cell maintenance, reproductive biology, and tumorigenesis.

Mounting evidence indicates that m^6^A modification and its regulatory enzymes not only shape intrinsic tumor cell functions but also profoundly influence the TME (15). As one of the major m^6^A demethylases, ALKBH5 has recently gained attention for its potential role in tumor immune regulation. On the one hand, ALKBH5 modulates m^6^A modification of immune-related transcripts in tumor cells—including cytokines/chemokines, antigen presentation molecules, and immune checkpoint regulators-thereby indirectly shaping immune cell recruitment, activation, and effector function (16). On the other hand, ALKBH5 in immune cells themselves (such as dendritic cells, macrophages, or T cells) can also regulate m^6^A programs of key molecules, influencing their differentiation, migration, and immune responsiveness. Thus, ALKBH5 exerts both tumor-intrinsic and immune-intrinsic effects, together orchestrating tumor immune evasion and responsiveness to immunotherapy (17). Mechanistically, ALKBH5 acts by demethylating specific mRNAs/lncRNAs/circRNAs, altering their interactions with m^6^A readers (such as YTH proteins or IGF2BPs), and thereby regulating transcript stability and translational efficiency (3). Its expression and activity are modulated by diverse cues, including hypoxia and inflammatory signals, positioning ALKBH5 as a key node that links microenvironmental factors to post-transcriptional immune regulation (18). Functionally, ALKBH5 activity can impact CD8^+^ T cell infiltration and cytotoxicity, the recruitment of myeloid-derived suppressor cells (MDSCs) and tumor-associated macrophage (TAM) subsets, as well as dendritic cell antigen presentation capacity, ultimately influencing the efficacy of immune checkpoint inhibitors (ICIs) and other anti-cancer immunotherapies (19).

Despite rapid progress, important gaps and controversies remain regarding the precise targets of ALKBH5 in distinct tumor types and immune cell lineages, its bidirectional effects (pro-immunogenic versus immunosuppressive), and its feasibility as a predictive biomarker or therapeutic target in clinical immunotherapy. This mini-review therefore focuses on the functional and mechanistic roles of ALKBH5 in tumor immune regulation. We aim to systematically summarize current evidence on how ALKBH5-mediated demethylation of transcripts affects immune-related pathways, to distinguish its tumor-intrinsic versus immune-intrinsic modes of action, to evaluate its potential impact on immunotherapy responsiveness, and to highlight key challenges for future research and clinical translation. Specifically, we will first overview the biological functions and upstream regulation of ALKBH5, then discuss evidence and mechanisms according to immune cell type and tumor–immune interactions, and finally explore its prospects as a therapeutic co-target and biomarker in immuno-oncology.

Previous reviews have primarily focused on the role of ALKBH5-mediated m6A modification in tumor progression. In contrast, this review places greater emphasis on the immunoregulatory functions of ALKBH5 within the tumor immune microenvironment. Moreover, we discuss in detail the upstream regulatory mechanisms governing ALKBH5 expression and activity, its functional heterogeneity across different cancer types, and the potential m6A-independent roles of ALKBH5, thereby providing a more comprehensive and updated perspective on its functions in cancer.

## 2 Biological functions of ALKBH5

### 2.1 m^6^A-dependent functions of ALKBH5

**Figure 1:**
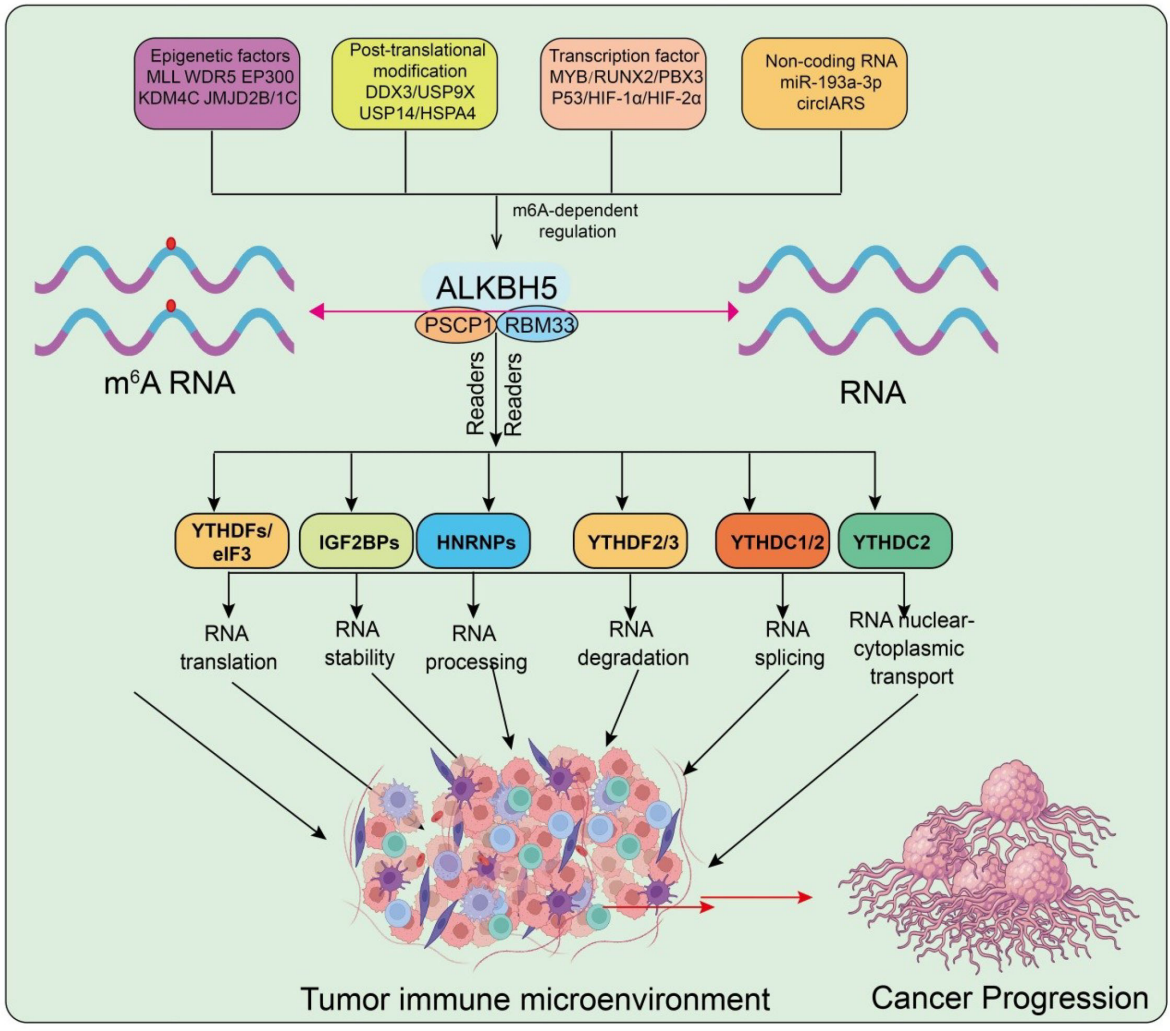
Regulation and functions of ALKBH5 in RNA metabolism and tumor immunity. Epigenetic factors, post-translational modifications, transcription factors, and non-coding RNAs regulate ALKBH5 expression and activity. ALKBH5 modulates m^6^A-dependent RNA metabolism, including RNA translation, stability, processing, degradation, splicing, and nucleocytoplasmic transport via different m^6^A readers (YTHDFs, IGF2BPs, HNRNPs, YTHDF2/3, YTHDC1/2). These processes collectively shape the tumor immune microenvironment and contribute to cancer progression. Red arrows indicate upregulation or downregulation.

As one of the key m^6^A demethylases, ALKBH5 plays a central role in the regulation of epitranscriptomic modifications (20). It selectively recognizes and removes m^6^A marks from target transcripts, thereby profoundly influencing RNA fate. The removal of m^6^A can alter transcript stability. For instance, by preventing the binding of decay-associated readers such as YTHDF2, leading to extended RNA half-life (21). In addition, ALKBH5 modulates alternative splicing by regulating methylation status at intron–exon junctions. Loss of ALKBH5 results in nuclear retention of certain transcripts, highlighting its critical function in RNA nuclear export (11). Through interactions with translation-promoting factors such as YTHDF1/3, ALKBH5 also regulates mRNA translational efficiency (**Figure 1****).** Collectively, the m^6^A-dependent functions of ALKBH5 span multiple stages of post-transcriptional regulation, from RNA processing to protein synthesis.

### 2.2 Regulation of ALKBH5 substrate specificity by RNA-binding proteins

Notably, the substrate specificity of ALKBH5 is not determined solely by its catalytic domains but is strongly influenced by cooperating RNA-binding proteins (RBPs). For example, YTH family proteins provide spatial guidance that enables ALKBH5 to recognize specific mRNAs more precisely, while IGF2BP proteins may compete or cooperate with ALKBH5 in target selection by stabilizing the same transcripts (22). This RBP-mediated network ensures that ALKBH5 activity is highly dependent on cell type and microenvironmental context. Recent studies further demonstrate that additional RBPs regulate the substrate selectivity and specificity of ALKBH5-mediated demethylation. For instance, acetylation of ALKBH5 at lysine 235 (K235), catalyzed by the acetyltransferase KAT8 and reversed by the deacetylase HDAC7, enhances its ability to recognize m^6^A-modified RNAs, thereby boosting demethylation activity. PSPC1 (23), acting as a regulatory subunit of ALKBH5, preferentially interacts with acetylated ALKBH5 at K235, facilitating its recruitment to m^6^A-marked transcripts and accelerating demethylation. Mitogenic signaling has been shown to induce K235 acetylation, which is elevated in tumors and promotes tumorigenesis (23). Thus, K235 acetylation and its cooperation with PSPC1 represent critical determinants of ALKBH5’s demethylase activity and oncogenic function. In parallel, our group recently identified RNA-binding motif protein 33 (RBM33) as a novel m^6^A-binding protein that forms a complex with ALKBH5 to mediate selective m^6^A demethylation. RBM33 recruits ALKBH5 to its m^6^A-marked targets and activates ALKBH5 by removing its SUMOylation (24). Functionally, RBM33 is indispensable for the progression of head and neck squamous cell carcinoma (HNSCC): by recruiting ALKBH5 to demethylate and stabilize DDIT4 mRNA, RBM33 promotes autophagy and thereby drives tumorigenesis (24) (**Figure 1**). The interaction between ALKBH5 and RBPs appears to contribute to its substrate selectivity, and current evidence suggests that this mechanism is largely tumor-specific rather than universal. For example, RBM33 has been reported to recruit ALKBH5 to specific mRNA targets in HNSC, whereas PSPC1 mediates a similar function in CRC. Such context-dependent interactions may reflect the heterogeneous expression patterns and cellular signaling environments across different tumor types.

### 2.3 Non–m^6^A-dependent functions of ALKBH5

In addition to its canonical role as an m^6^A demethylase, ALKBH5 also exerts several non–m^6^A-dependent functions (25). On the one hand, studies suggest that it may participate in the regulation of other RNA modifications, such as m¹A, or modulate the stability and localization of noncoding RNAs through direct binding (26). On the other hand, ALKBH5 itself can act as an RNA-binding protein, contributing to the formation of stress granules and the subcellular localization of RNAs. Moreover, ALKBH5 is capable of interacting with transcription factors such as HIF-1α to regulate the expression of hypoxia-responsive genes, a function independent of its demethylase activity. Recent research has shown that ALKBH5 expression is elevated under conditions of obesity or diabetes. In these states, it undergoes phosphorylation at serine 362 (S362) via the glucagon–PKA signaling pathway, leading to its translocation from the nucleus to the cytoplasm. In the cytoplasm, ALKBH5 binds to the m^6^A-modified region of Gcgr mRNA and removes the methyl mark, thereby stabilizing Gcgr transcripts. This enhances glucagon receptor (GCGR) signaling and promotes hepatic gluconeogenesis. Conversely, knockout or mutation of ALKBH5 suppresses GCGR signaling, lowers blood glucose levels, and confers resistance to diet-induced hyperglycemia in mice. ALKBH5 also regulates lipid metabolism in a demethylase-independent manner (27). Two loop regions in its structure (Q145–G152 and C231–E242) directly bind to the enhancer of the Egfr gene, thereby promoting epidermal growth factor receptor (EGFR) transcription. This activation of the EGFR–PI3K-AKT-mTORC1 signaling axis drives lipid synthesis. Inhibition of ALKBH5 reduces the activity of this pathway, improving fatty liver and hyperlipidemia (27). Gene therapy strategies targeting ALKBH5, such as AAV-shRNA or GalNAc-siRNA, have been successfully applied in diabetic mouse models, leading to a marked reduction of ALKBH5 levels in the liver (27). This intervention significantly ameliorated hyperglycemia, hyperlipidemia, and hepatic steatosis, outperforming conventional treatment approaches. Collectively, these findings demonstrate that ALKBH5 is not merely a “demethylase” but rather a multidimensional regulator of RNA metabolism and cellular homeostasis, with implications extending from tumor biology to metabolic diseases. Increasing studies have indicated that ALKBH5 may perform certain regulatory functions beyond its canonical m6A demethylase activity. These m6A-independent effects remain a subject of debate and could be attributed to ALKBH5’s potential roles in RNA metabolism, chromatin regulation, or protein–protein interactions. Further research is needed to clarify the molecular basis and physiological significance of these noncanonical functions.

### 2.4 Upstream regulatory mechanisms of ALKBH5 expression

The expression and activity of ALKBH5 are subject to multilayered upstream regulation, particularly in cancer. At the epigenetic level, histone modifications represent key determinants of ALKBH5 transcriptional activity. Factors such as MLL, WDR5, and EP300 enhance ALKBH5 transcription by promoting activating histone marks (28). Conversely, loss of LKB1 induces hypermethylation of 5mC at the CTCF-binding site in the ALKBH5 promoter, preventing CTCF occupancy and concomitantly increasing active histone modifications, thereby upregulating ALKBH5 expression. In contrast, histone demethylases such as KDM4C and JMJD2B/1C contribute to transcriptional repression of ALKBH5 (29). In addition, the HPV E7 oncoprotein can enhance ALKBH5 expression through E2F1-mediated histone modifications (H3K27ac and H3K4me3) as well as DDX3-mediated post-transcriptional regulation (30). At the transcription factor level, multiple regulators directly bind to the promoter or enhancer of ALKBH5. Transcriptional activators such as MYB, RUNX2, and PBX3 upregulate ALKBH5, whereas the tumor suppressor p53 represses its transcription. Furthermore, hypoxia-inducible factors HIF-1α and HIF-2α strongly activate ALKBH5 transcription in hypoxic tumor microenvironments, highlighting the importance of microenvironmental cues (31, 32). In terms of noncoding RNA regulation, long noncoding RNAs (lncRNAs) often act as molecular scaffolds to facilitate the interaction of ALKBH5 with its target mRNAs, thereby enhancing its functional activity. miRNAs also participate in this regulatory network; for instance, miR-193a-3p binds to the 3′UTR of ALKBH5 mRNA to promote its degradation, whereas ALKBH5 in turn suppresses the maturation of miR-193a-3p through demethylation, forming a negative feedback loop (33). In ovarian cancer cells co-cultured with M2-type macrophages, activation of the TLR4 signaling pathway upregulates ALKBH5. The RNA helicase DDX3 binds to ALKBH5 and enhances its demethylation activity (34). Our group demonstrated that reactive oxygen species (ROS) can modulate ALKBH5 through post-translational modifications (PTMs), leading to a global increase in m^6^A levels and rapid induction of thousands of genes involved in DNA damage repair and other processes (35). Mechanistically, ROS activate the ERK/JNK signaling pathway, promoting SUMOylation of ALKBH5, which obstructs substrate accessibility and inhibits its demethylase activity (35). In addition, deubiquitinating enzymes play a critical role in ALKBH5 stability. USP9X removes K48-linked polyubiquitin chains at lysine 57 (K57) of ALKBH5, thereby stabilizing its protein level and promoting AML cell survival (36). Similarly, USP14 prevents HECW2-mediated K48-linked ubiquitination and degradation of ALKBH5 (37, 38). MST4 kinase phosphorylates ALKBH5 at serine 64 and 69, strengthening its interaction with USP14 and facilitating deubiquitination. In gastric cancer (GC) tissues, histone acetylation induces upregulation of HSPA4, which in turn enhances the protein stability of ALKBH5 (39). Collectively, these findings highlight that the upstream regulatory network of ALKBH5 is highly complex, encompassing epigenetic modifications, transcription factors, noncoding RNAs, inflammatory signaling pathways, and post-translational modifications. Together, these layers of regulation shape the biological functions of ALKBH5 in tumorigenesis and cancer progression (**Figure 1**).

## 3 Roles of ALKBH5 in the TME

**Figure 2:**
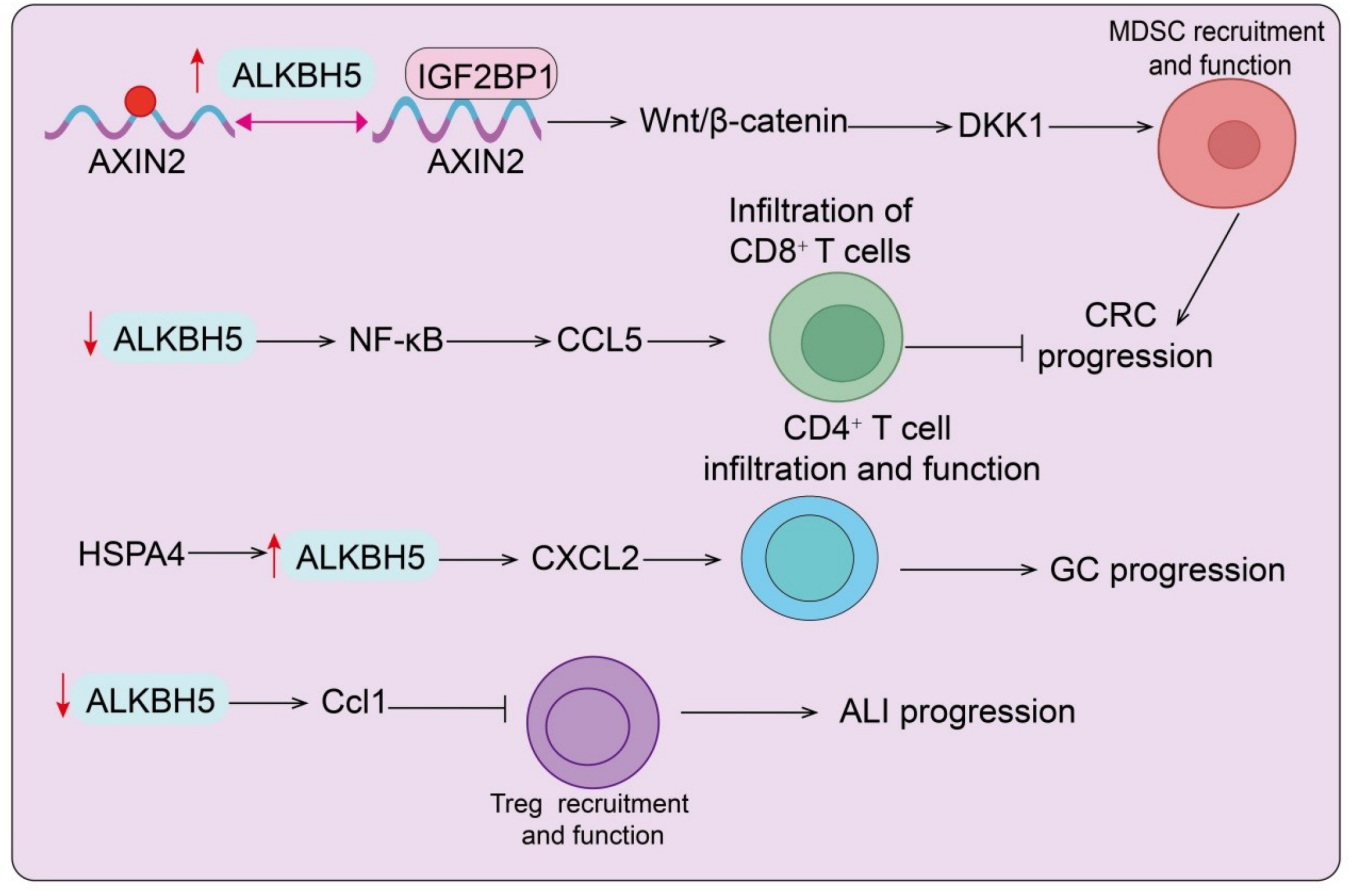
Role of ALKBH5 in regulating T cell infiltration and tumor progression. ALKBH5 controls immune cell infiltration and tumor development through multiple pathways:ALKBH5/IGF2BP1/AXIN2 axis activates Wnt/β-catenin signaling, leading to MDSC recruitment and CRC progression. Reduced ALKBH5 activates NF-κB/CCL5 signaling, impairing CD4^+^ T cell infiltration and function. ALKBH5/HSPA4/CXCL2 axis promotes GC progression. Downregulation of ALKBH5 decreases Ccl1 expression, reducing Treg recruitment and contributing to ALI progression. Red arrows indicate upregulation or downregulation.

**Figure 3:**
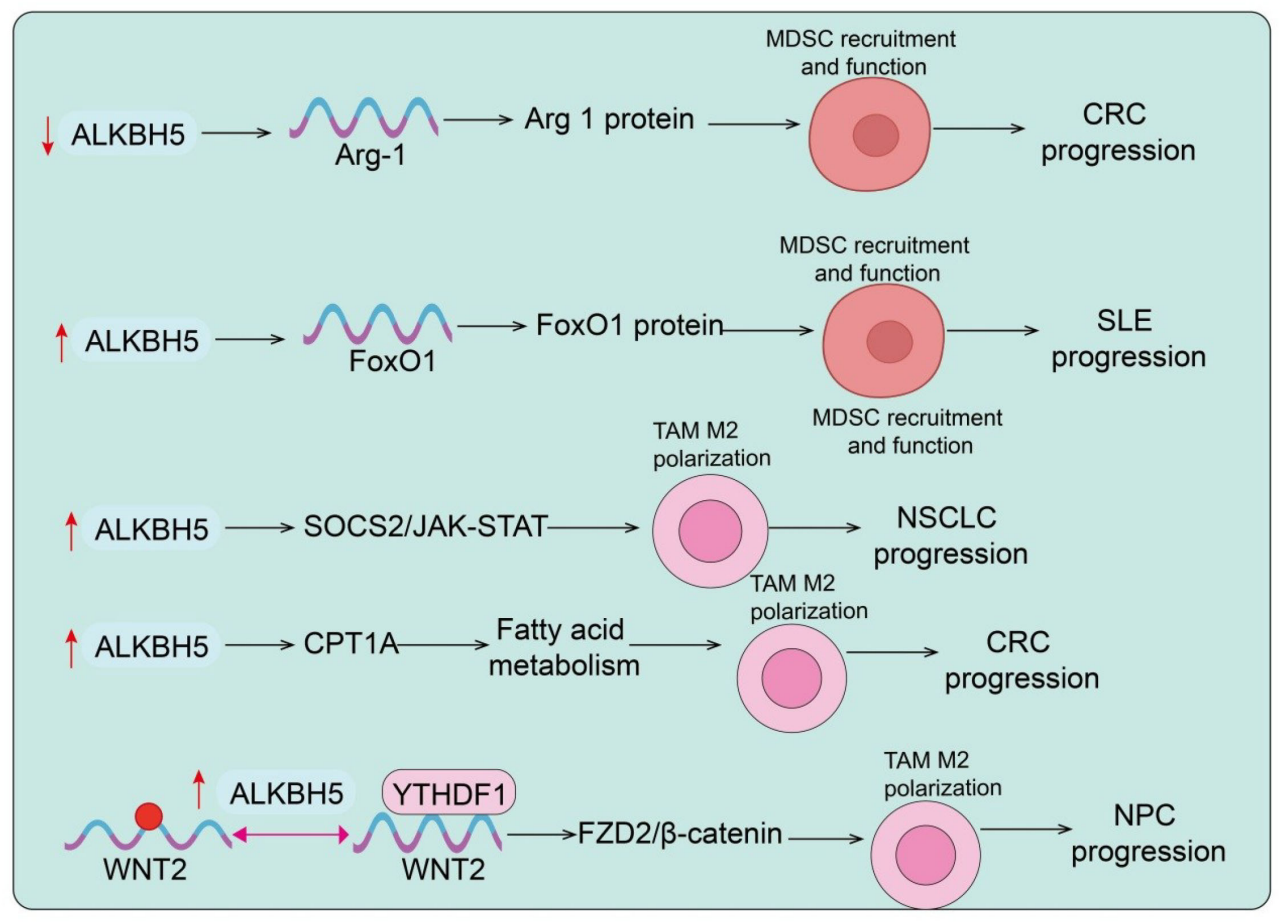
ALKBH5 regulates MDSC recruitment and TAM polarization in cancer and immune diseases. Reduced ALKBH5 increases Arg-1 expression, enhancing MDSC recruitment and CRC progression. Upregulated ALKBH5 stabilizes FoxO1, promoting MDSC recruitment and SLE progression. ALKBH5/SOCS2/JAK-STAT signaling enhances TAM M2 polarization, contributing to NSCLC progression. ALKBH5 promotes fatty acid metabolism via CPT1A, enhancing TAM M2 polarization and CRC progression. ALKBH5/YTHDF1/WNT2 axis promotes TAM M2 polarization, leading to NPC progression. Red arrows indicate upregulation or downregulation.

**Figure 4:**
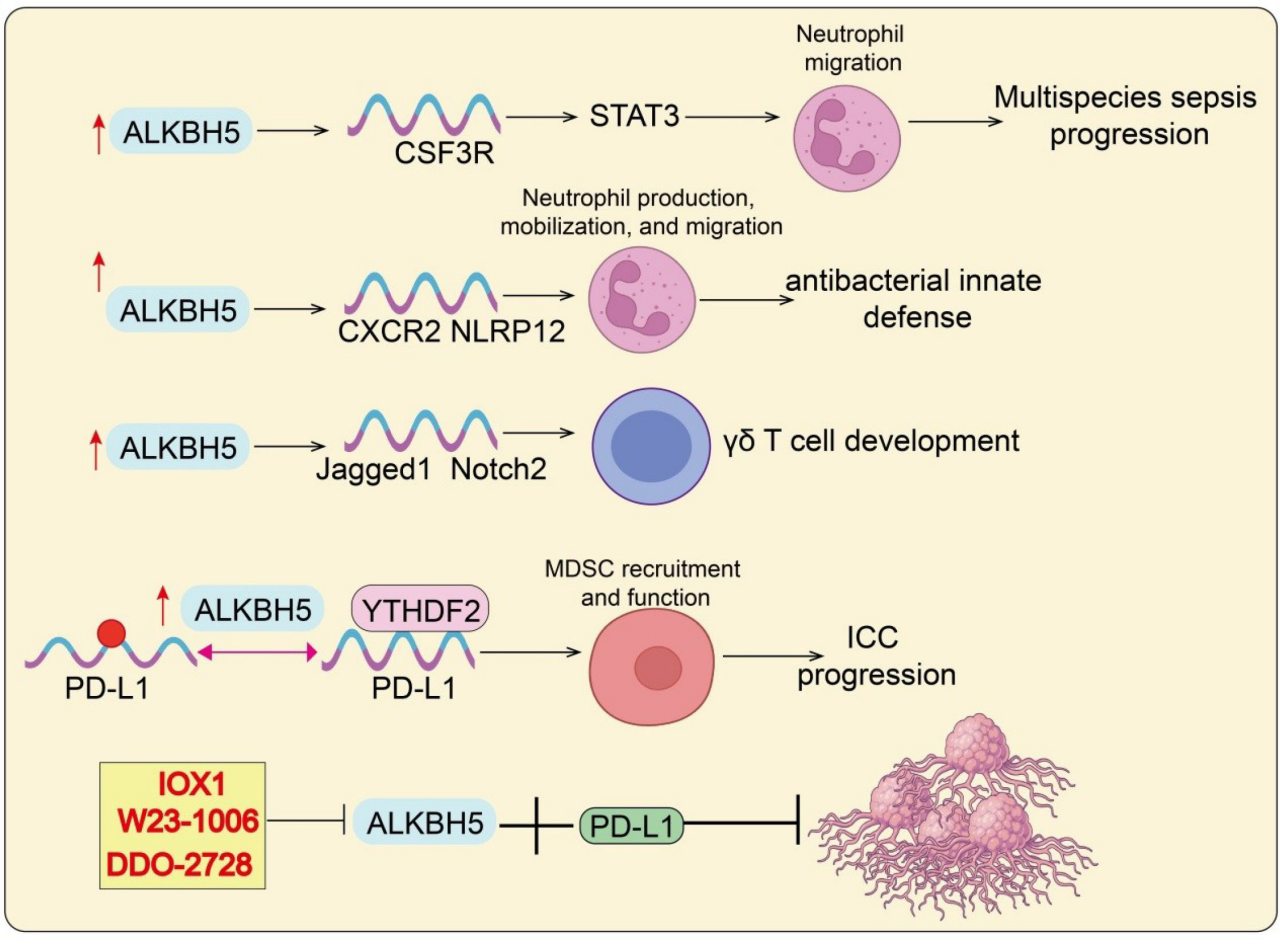
ALKBH5-mediated regulation of immune cell functions in infection, inflammation, and cancer. ALKBH5/CSF3R/STAT3 axis promotes neutrophil migration and contributes to sepsis progression. ALKBH5 upregulates CXCR2 and NLRP12, enhancing antibacterial innate defense. ALKBH5/Jagged1/Notch2 axis regulates γδ T cell development. ALKBH5/YTHDF2/PD-L1 pathway promotes MDSC recruitment and ICC progression. ALKBH5 inhibitors (IOX1, W23-1006, DDO-2728) suppress PD-L1 expression, thereby attenuating tumor immune evasion. Red arrows indicate upregulation or downregulation. ALI, acute lung injury; CRC, colorectal cancer; GC, gastric cancer; ICC, intrahepatic cholangiocarcinoma; MDSC, myeloid-derived suppressor cell; NPC, nasopharyngeal carcinoma; NSCLC, non-small cell lung cancer; SLE, systemic lupus erythematosus; TAM, tumor-associated macrophage.

The TME plays a central role in determining tumor initiation, progression, immune evasion, and therapeutic response (40). As a critical m^6^A demethylase, ALKBH5 profoundly influences the crosstalk between tumor cells and diverse immune cell subsets through modulation of RNA methylation dynamics. In recent years, accumulating evidence has revealed that ALKBH5 regulates not only the infiltration and effector functions of lymphocytes such as CD8^+^ T cells, CD4^+^ T cells, and Tregs, but also the recruitment and polarization of innate immune cells including MDSCs, TAMs, and neutrophils. Moreover, ALKBH5 shapes either immunosuppressive or immune-activating milieus by modulating the expression of key chemokines, cytokines, and metabolic pathways, thereby dictating the strength of antitumor immune responses and the sensitivity to immunotherapies (41). Importantly, the function of ALKBH5 in the TME appears to be context-dependent and may exert dual effects (41). While ALKBH5 often promotes immunosuppression and tumor progression, under certain tumor types or immune settings it may enhance antitumor immunity. The immunoregulatory effects of ALKBH5 appear to involve both tumor-intrinsic and immune cell–driven mechanisms. In tumor cells, ALKBH5 modulates the expression of chemokines, cytokines, and immune checkpoint molecules, thereby influencing immune cell infiltration and immune evasion. In immune cells such as macrophages and T cells, ALKBH5 regulates differentiation, effector function, and metabolic adaptation. Therefore, the overall impact of ALKBH5 on tumor immunity likely reflects a dynamic interplay between tumor and immune compartments. Taken together, systematically elucidating the multifaceted roles of ALKBH5 within the TME is essential for advancing our understanding of tumor immune regulation and for developing novel, precision-based therapeutic strategies (**Figures 2**–**4**).

### 3.1 Regulation of T cell infiltration and function

T cells are the central effector cells of antitumor immunity, and their degree of infiltration and functional state directly determine the immune activity within the TME (42). In colorectal cancer (CRC), high ALKBH5 expression predicts poor prognosis. ALKBH5 promotes the accumulation of myeloid-derived suppressor cells (MDSCs) while reducing the infiltration of natural killer (NK) cells and cytotoxic CD8^+^ T cells, thereby facilitating colorectal tumorigenesis in syngeneic models, CD34^+^ humanized mice, and intestinal Alkbh5 knock-in mice (43). Mechanistically, AXIN2, a Wnt pathway inhibitor, has been identified as the key downstream target of ALKBH5. ALKBH5 binds to and demethylates AXIN2 mRNA, leading to its dissociation from the m^6^A reader IGF2BP1 and subsequent degradation, resulting in hyperactivation of the Wnt/β-catenin signaling pathway. Consequently, ALKBH5 upregulates Wnt/β-catenin target genes, including Dickkopf-related protein 1 (DKK1). ALKBH5-induced DKK1 drives MDSC recruitment and immune suppression in CRC, which can be abrogated by anti-DKK1 blockade both *in vitro* and *in vivo (*43). Furthermore, ALKBH5-targeted siRNA delivered via vesicle-like nanoparticles or anti-DKK1 treatment enhances the efficacy of anti-PD-1 therapy by promoting antitumor immunity and restraining CRC growth (43) (**Figure 2**). Conversely, other studies reported downregulation of ALKBH5 expression in CRC, with low levels significantly associated with poor overall survival (OS). Functionally, ALKBH5 overexpression inhibited CRC cell proliferation, migration, and invasion, whereas its silencing exerted opposite effects. Mechanistically, ALKBH5 suppressed the NF-κB signaling pathway, thereby reducing CCL5 expression and promoting CD8^+^ T cell infiltration into the CRC microenvironment (44) (**Figure 2**). In gastric cancer (GC), histone acetylation upregulated HSPA4, which enhanced ALKBH5 protein stability. ALKBH5, in turn, downregulated CD58 expression via m^6^A-dependent regulation in GC cells. Co-culture of CD8^+^ T cells with HSPA4-overexpressing GC cells impaired T cell cytotoxicity and activated the PD-1/PD-L1 axis (39). Clinically, in patients receiving surgery alone, HSPA4 upregulation correlated with poorer 5-year OS and served as an independent prognostic factor. Interestingly, among GC patients treated with chemotherapy combined with anti-PD-1 therapy, responders exhibited significantly higher HSPA4 upregulation compared with non-responders (39). With regard to CD4^+^ T cells, ALKBH5-but not FTO, was shown to maintain the capacity of naïve CD4^+^ T cells to induce graft-versus-host colitis. Conditional deletion of ALKBH5 in T cells conferred protection in experimental autoimmune encephalomyelitis (EAE) (**Figure 2**). During induced neuroinflammation, ALKBH5 loss increased m^6^A modification on interferon-γ (IFN-γ) and chemokine CXCL2 mRNAs, thereby reducing their stability and protein expression (45). These changes attenuated CD4^+^ T cell effector functions and limited neutrophil recruitment into the central nervous system. Collectively, ALKBH5 regulates the infiltration and functional activity of both CD8^+^ and CD4^+^ T cells through multiple molecular pathways (45). Its roles appear context-dependent, exerting either immunosuppressive or immune-enhancing effects across different cancer types and immune environments, thereby profoundly influencing tumor progression and responses to immunotherapy.

### 3.2 ALKBH5 function and mechanism in Tregs

Tregs play a pivotal role in maintaining immune homeostasis and establishing an immunosuppressive microenvironment, and their abundance and function are tightly regulated by RNA-modifying enzymes (46). In mouse models, ALKBH5 expression was markedly upregulated in lung tissues following lipopolysaccharide (LPS) exposure. Functional assays demonstrated that Alkbh5 deficiency attenuated LPS-induced acute lung injury (ALI), characterized by reduced serum markers of lung injury (47), diminished immune cell infiltration, fibrosis, and apoptosis, whereas Alkbh5 overexpression exacerbated pathological damage. *In vitro*, Alkbh5 knockdown enhanced the viability of alveolar epithelial MLE12 cells and reduced apoptosis and ROS production. Mechanistically, ALKBH5 directly bound to and degraded Ccl1 mRNA, thereby impairing Ccl1-mediated Treg recruitment and facilitating the progression of ALI(**Figure 2**). Interventional studies further revealed that either pharmacological inhibition of Alkbh5 using the antagonist DDO-2728 or supplementation with recombinant Ccl1 protein promoted Treg infiltration, improved histopathological features, and alleviated lung injury (47). Collectively, these findings suggest that ALKBH5 regulates Treg recruitment by modulating Ccl1 levels, thereby reshaping the immunosuppressive microenvironment. This highlights the dual relevance of ALKBH5 in both inflammatory diseases and tumor immune evasion.

### 3.3 The role of ALKBH5 in MDSCs recruitment and functional

MDSCs are critical regulators in the progression of various diseases, and their recruitment and immunosuppressive activity are closely modulated by the m^6^A demethylase ALKBH5 (48). In CRC mouse models, MDSCs exhibited elevated m^6^A levels concomitant with downregulation of ALKBH5. Functional assays revealed that ALKBH5 overexpression attenuated the immunosuppressive capacity and tumor-promoting activity of MDSCs. Mechanistically, ALKBH5 decreased the m^6^A modification of Arg-1 mRNA, thereby reducing its stability and protein expression, suggesting that ALKBH5 loss in CRC may enhance Arg-1–mediated immunosuppression and tumor progression(**Figure 3**). Conversely, other studies reported high ALKBH5 expression in CRC, which correlated with poor prognosis (49). In syngeneic tumor models, CD34^+^ humanized mice, and intestine-specific Alkbh5 transgenic mice, ALKBH5 promoted MDSC accumulation while diminishing NK and CD8^+^ T-cell infiltration, thereby driving tumorigenesis. This effect was mediated by ALKBH5-dependent demethylation of AXIN2 mRNA, leading to its degradation and hyperactivation of the Wnt/β-catenin pathway (43). Upregulation of the Wnt target gene DKK1 subsequently recruited MDSCs and reinforced immunosuppression. Notably, ALKBH5 deletion sensitized tumors to immunotherapy by modulating Mct4/Slc16a3 expression and lactate metabolism, reshaping Treg and MDSC composition in the TME (41). Small-molecule inhibitors of ALKBH5 significantly enhanced immunotherapy efficacy, and clinical data further linked ALKBH5 expression with immunotherapeutic responsiveness in melanoma patients. Beyond cancer, ALKBH5 also modulates MDSC function in autoimmune diseases (50). In imiquimod-induced lupus mouse models, Shuangshen Fang (SF) alleviated disease symptoms and reduced M-MDSC accumulation in blood and spleen. Mechanistically, SF upregulated FoxO1, enhancing M-MDSC–mediated suppression of Th17 cells, whereas myeloid-specific FoxO1 deletion abrogated this effect. The key SF component delphinidin chloride (DP) stabilized FoxO1 mRNA by inhibiting ALKBH5-mediated m^6^A demethylation, thereby augmenting MDSC immunosuppression (50) (**Figure 3**). In systemic lupus erythematosus (SLE) patients and lupus mice, M-MDSCs displayed reduced FoxO1 expression, and FoxO1 deficiency exacerbated B-cell dysfunction by activating the Met/COX2/PGE2 axis. Pharmacological inhibition of Met (e.g., capmatinib) ameliorated lupus progression (50). Collectively, ALKBH5 enhanced FoxO1 mRNA stability by reducing m^6^A modification, thereby improving disease outcomes. Taken together, ALKBH5 exerts context-dependent effects on MDSCs: it promotes tumor-associated immunosuppression via the Wnt/β-catenin-DKK1 axis, yet modulates autoimmune responses by stabilizing FoxO1 mRNA and regulating MDSC–T/B cell interactions. These findings underscore the dual role of ALKBH5 in tumor immune evasion and autoimmune regulation, highlighting its potential as a therapeutic target.

### 3.4 The role of ALKBH5 in TAM recruitment and functional

TAMs are central players in shaping the immunological landscape of the TME and promoting tumor progression (51). Accumulating evidence indicates that ALKBH5 is a key regulator of TAM recruitment and polarization. In non-small cell lung cancer (NSCLC), cigarette smoke induced the accumulation of M2-type TAMs, which secreted EVs enriched with circEML4 (52). These EVs transferred circEML4 into tumor cells, where it interacted with ALKBH5, restricted its nuclear localization, and increased global m^6^A levels, thereby promoting malignant progression via the SOCS2/JAK-STAT pathway (52) (**Figure 3**). Similarly, in CRC, ALKBH5 facilitated macrophage M2 polarization, enhancing CRC cell viability, proliferation, and migration. Mechanistically, ALKBH5 stabilized CPT1A expression by demethylation, promoting fatty acid oxidation and energy metabolism to sustain M2 polarization and tumor growth (53). In NPC, ALKBH5 cooperated with YTHDF1 to stabilize WNT2 mRNA and elevate WNT2 protein levels, which not only promoted M2 polarization of TAMs but also enhanced tumor cell proliferation and metastasis via autocrine activation of the FZD2/β-catenin axis, establishing a vicious cycle (54). Beyond cancer, ALKBH5 also regulates macrophage functions in metabolic diseases. In atherosclerosis, ALKBH5 was upregulated in patient and murine arterial plaques. Macrophage-specific deletion of ALKBH5 reduced foam cell formation, inhibited senescence-associated secretory phenotype (SASP), and increased plaque stability. Mechanistically, ALKBH5 stabilized CCL5 mRNA, thereby activating the CCL5/CCR5/autophagy pathway, which promoted foam cell senescence and recruited CD8^+^IFNγ^+^ T cells, exacerbating inflammation (44). In hepatocellular carcinoma (HCC), high ALKBH5 expression correlated with poor prognosis. Mechanistic studies revealed that ALKBH5 upregulated MAP3K8 expression in an m^6^A-dependent manner, thereby activating JNK/ERK signaling and enhancing IL-8 secretion, which recruited PD-L1^+^ macrophages to foster tumor growth, metastasis, and immune evasion (55). In summary, ALKBH5 regulates TAM recruitment and M2 polarization across diverse disease models through mechanisms involving metabolic reprogramming, cytokine secretion, and activation of oncogenic signaling pathways. These findings position ALKBH5 as both a facilitator of tumor immune escape and a modulator of inflammatory balance in metabolic diseases, underscoring its diagnostic and therapeutic potential.

### 3.5 Neutrophils

ALKBH5 plays a pivotal role in the generation, mobilization, and migration of neutrophils. In a cecal ligation and puncture (CLP)-induced sepsis model, loss of ALKBH5 resulted in impaired production of immature neutrophils in the bone marrow, retention of mature neutrophils within the marrow, and reduced release into the peripheral blood, leading to a marked reduction of neutrophils at the infection site (56). Mechanistically, ALKBH5 enhances the stability of CSF3R mRNA by removing its m^6^A modification, thereby increasing G-CSFR expression and activating STAT3 signaling to sustain neutrophil mobilization. However, during bacterial infection, the binding affinity of ALKBH5 to CSF3R mRNA decreases, causing downregulation of G-CSFR and further impairing mobilization (56) (**Figure 4**). In addition, ALKBH5 is indispensable for neutrophil migration. ALKBH5-deficient mice in the CLP model exhibited elevated bacterial burden, increased inflammatory cytokine levels, and higher mortality, attributable to impaired neutrophil trafficking. Mechanistic studies revealed that ALKBH5 modulates the expression of migration-related molecules via m^6^A demethylation: upregulating migration-promoting factors (CXCR2, NLRP12) while downregulating migration-inhibitory factors (PTGER4, TNC, WNK1) (45). Collectively, ALKBH5 governs neutrophil biogenesis, mobilization, and migration, and its loss severely compromises host antibacterial defense and exacerbates sepsis progression.

### 3.6 γδ T cells

γδ T cells are abundantly distributed in mucosal tissues and play essential roles in immune surveillance and tissue homeostasis (42). Although they originate from the thymus, the fine-tuned mechanisms regulating their development remain unclear. Recent studies revealed that ALKBH5 deficiency significantly promotes the expansion of γδ T cells and enhances host resistance to intestinal Salmonella infection (42). Mechanistically, loss of ALKBH5 elevates m^6^A modification levels in thymocytes, leading to downregulation of key molecules in the Notch signaling pathway, including Jagged1 and Notch2. Impaired Jagged1/Notch2 signaling consequently promotes the proliferation and differentiation of γδ T cell precursors, resulting in an expanded pool of mature γδ T cells (57) (**Figure 4**). In summary, ALKBH5 functions as a negative regulator and developmental “checkpoint” in early γδ T cell differentiation. Its loss reshapes the γδ T cell repertoire and enhances immune potential, offering new insights into the developmental mechanisms and functional regulation of γδ T cells.

## 4 ALKBH5 and immunotherapy

### 4.1 Synergy with immune checkpoint inhibitors

As a key m^6^A demethylase, ALKBH5 modulates PD-L1 expression and reshapes the TME, thereby influencing immune evasion and therapeutic response. In intrahepatic cholangiocarcinoma, ALKBH5 directly binds and demethylates PD-L1 mRNA, reducing m^6^A modification in its 3′UTR, which prevents YTHDF2-mediated degradation, stabilizes PD-L1 expression, and suppresses T cell expansion and cytotoxicity (58). Histological and single-cell mass cytometry analyses further demonstrated that ALKBH5 not only enhances PD-L1 expression in monocytes/macrophages but also promotes infiltration of MDSC-like cells, highlighting its complex regulatory role in the TME. Clinically, strong nuclear expression of ALKBH5 was associated with better responses to anti–PD-1 therapy, suggesting its potential as a predictive biomarker for immunotherapy outcomes. In NSCLC, ALKBH5 expression levels correlate with PD-L1 expression, macrophage infiltration, and immunotherapy response (59). Mechanistically, ALKBH5 promotes activation of the JAK2/p-STAT3 pathway in an m^6^A-dependent manner, facilitating tumor progression (59). Concurrently, ALKBH5 induces secretion of CCL2 and CXCL10, recruiting PD-L1^+^ M2-like TAMs, and synergizes with TAM-derived IL-6 to amplify JAK2/p-STAT3 signaling (59). Together, these findings reveal that ALKBH5 promotes immune escape and tumor progression through dual mechanisms: stabilizing PD-L1 in tumor cells and remodeling the TAM-driven immunosuppressive TME. Targeting ALKBH5 may therefore enhance the efficacy of anti–PD-1/PD-L1 therapy and serve as a clinically relevant predictive biomarker.

### 4.2 Potential of ALKBH5 inhibitors in combination immunotherapy

Emerging studies highlight the therapeutic potential of targeting ALKBH5 to reprogram the TME and augment immunotherapy responses (60). In ovarian cancer, NGR-modified biomimetic nanovesicles (NGR-BNVs) achieved efficient delivery of ALKBH5 siRNA, markedly suppressing tumor proliferation and inducing apoptosis. In murine models, silencing ALKBH5 not only inhibited tumor growth and metastasis but also promoted CD8^+^ T cell infiltration while reducing the proportion of Tregs and MDSCs, thereby reversing immune resistance via TME modulation (61). Similarly, in glioblastoma, ALKBH5 promoted immune evasion by regulating PD-L1 expression. Genetic deletion or pharmacological inhibition of ALKBH5 (e.g., IOX1) significantly enhanced the efficacy of anti–PD-1 therapy, resulting in tumor regression, increased infiltration of cytotoxic lymphocytes, and elevated proinflammatory cytokines, supporting the preclinical feasibility of ALKBH5 inhibition combined with ICIs (62). In parallel, novel ALKBH5 inhibitors are being developed. Structure-based virtual screening identified a selective covalent inhibitor, W23-1006, which forms a covalent bond with residue C200 (63). Its inhibitory potency is ~30-fold and ~8-fold greater than that against FTO and ALKBH3, respectively, and it markedly suppressed proliferation, migration, and xenograft growth of triple-negative breast cancer. Another inhibitor, DDO-2728, demonstrated high selectivity and safety; in acute myeloid leukemia, it increased m^6^A modification, reduced TACC3 mRNA stability, and induced cell cycle arrest, thereby effectively suppressing tumor progression (64). Additionally, a series of maleimide derivatives targeting the noncatalytic C200 site were developed; compound 18l exhibited potent inhibitory activity (IC_50_ = 0.62 μM), robust anti-leukemic effects, and achieved a 66.3% tumor inhibition rate in NB4 xenograft models (65). Collectively, ALKBH5 inhibitors not only demonstrate direct antitumor activity but also downregulate PD-L1, enhance effector T cell function, and suppress infiltration of immunosuppressive cells, thereby exhibiting synergistic potential with ICIs. These findings provide solid experimental and translational foundations for ALKBH5 inhibitors in combination immunotherapy(**Figure 4**). Because ALKBH5 participates in essential physiological processes, distinguishing its normal versus malignant functions is critical for safe targeting. Approaches to increase specificity include exploiting differences in expression levels or interacting partners between tumor and normal tissues, designing inhibitors that disrupt tumor-specific protein–protein interactions or post-translationally modified forms of ALKBH5, and employing tumor-restricted delivery systems (for example, ligand-directed nanoparticles or antibody–drug conjugates). In parallel, predictive biomarkers and comprehensive preclinical toxicology using tissue-specific knockout models will be needed to define therapeutic windows and avoid broad cytotoxicity.

### 4.3 Precision medicine perspectives: ALKBH5 as a predictive biomarker of immunotherapy response

With the rapid development of immunotherapy strategies such as ICIs, identifying patients who are sensitive or resistant to treatment has become a central challenge in precision medicine. Recent evidence suggests that ALKBH5 expression levels and enzymatic activity may serve as predictive biomarkers of immunotherapy response. On one hand, ALKBH5 regulates m^6^A demethylation to influence the stability and translation of immune-related genes, including PD-L1, chemokines, and cytokines, thereby shaping the TME (59, 66). On the other hand, ALKBH5 activity correlates with distinct immune cell infiltration patterns, potentially determining patient sensitivity to immunotherapy. Clinical cohort studies and transcriptomic analyses indicate that high ALKBH5 expression is often associated with poor response rates to immunotherapy, suggesting its role in establishing an immunosuppressive microenvironment. Conversely, other evidence suggests that ALKBH5 deficiency or low expression may promote immune escape mechanisms, indicating that its predictive value may be context- and cancer-type–dependent. Therefore, future studies are needed to validate the robustness and generalizability of ALKBH5 as a biomarker in larger, multi-cancer clinical cohorts, integrating multi-omics data with immunotherapy follow-up. If validated, ALKBH5 assessment could not only facilitate patient stratification and therapeutic prediction but also complement existing biomarkers (e.g., TMB, PD-L1 expression, TIL levels), thereby advancing precision immunotherapy strategies.

## 5 Discussion and perspectives

Current evidence suggests that ALKBH5 may play dual roles in tumor immune regulation, either enhancing antitumor immune responses or promoting tumor immune escape by shaping an immunosuppressive microenvironment. For example, ALKBH5-mediated m^6^A demethylation can stabilize transcripts encoding immune-activating factors, thereby strengthening effector T cell functions. Conversely, in different contexts, ALKBH5 may also stabilize immune checkpoint molecules or chemokines, facilitating the recruitment of Tregs or MDSCs, which dampens antitumor immunity. A key challenge lies in defining the directionality of ALKBH5’s immune effects across distinct tissue environments and cancer types (43).

Mechanistically, ALKBH5 exerts both m^6^A-dependent and independent functions in immune regulation. On one hand, it reshapes immune pathways by altering the stability and translation of target mRNAs in an m^6^A-dependent manner. On the other, it acts in an m^6^A-independent manner-for instance, functioning as an RNA-binding protein to modulate transcript subcellular localization or interacting with transcription factors to influence signaling cascades (27). Recent studies have shown that ALKBH5 is specifically upregulated in lung cancer tissues, and its expression is strongly associated with poor prognosis and infiltration of PD-L1^+^ TAMs. Intriguingly, dual regulatory mechanisms have been uncovered: (i) an m^6^A-dependent ALKBH5–YTHDF2–JAK2 axis modulating JAK-STAT signaling (52, 59), and (ii) a nonenzymatic pathway through CCL2/CXCL10 regulation affecting immune cell recruitment and polarization (59). The substrate selectivity of ALKBH5 is constrained and guided by RNA-binding protein networks, which may explain its cell type– and context-specific functions. Furthermore, although traditionally classified as a post-transcriptional regulator, emerging data suggest that ALKBH5 might indirectly influence chromatin dynamics and 3D genome architecture-an exciting frontier requiring further validation. Despite rapid progress, studies on ALKBH5 in tumor immunity remain limited. First, many findings are derived from murine models, lacking sufficient validation in human tissues and clinical datasets. Second, most work has been restricted to single cancer types, without cross-cancer systematic comparisons. Third, comprehensive analyses of ALKBH5’s direct targets, regulatory networks, and immune cell–specific effects are still missing (67). In addition to ALKBH5, FTO is another m6A demethylase involved in tumor immunity. Although both enzymes modulate the immune response through m6A-dependent mechanisms, accumulating evidence suggests that ALKBH5 exerts a more prominent role in shaping the tumor immune microenvironment, particularly by regulating immune checkpoint expression and T cell infiltration. In addition to ALKBH5, FTO is another m6A demethylase involved in tumor immunity. Although both enzymes modulate the immune response through m6A-dependent mechanisms, accumulating evidence suggests that ALKBH5 exerts a more prominent role in shaping the tumor immune microenvironment, particularly by regulating immune checkpoint expression and T cell infiltration.

Future research should focus on three directions. First, applying high-throughput sequencing and single-cell multi-omics to systematically map ALKBH5’s immune-related transcriptome targets and regulatory networks, with particular emphasis on cell type–specific effects. Second, developing more selective and pharmacokinetically favorable ALKBH5 inhibitors and investigating their immunomodulatory functions in tumor models. Third, advancing the combination of ALKBH5 targeting with ICIs, evaluating their synergistic potential in preclinical and clinical studies to enhance immunotherapy response rates and efficacy.

ALKBH5 plays roles in both innate and adaptive immunity, but its functions are not entirely overlapping. In innate immunity, ALKBH5 mainly regulates antiviral responses, inflammatory cytokine production, and macrophage or dendritic‐cell activation by controlling mRNA stability and translation. In adaptive immunity, its effects are more focused on T-cell differentiation, activation, and exhaustion through modulation of key immune-regulatory transcripts. Thus, while ALKBH5’s epitranscriptomic regulation is fundamental to both arms of the immune system, the specific targets and biological outcomes differ.

Current research on ALKBH5 in tumor immunity has revealed its importance, but several limitations and unresolved questions remain. First, mechanistic understanding is still incomplete: although ALKBH5 has been linked to changes in immune infiltration and cytokine production, many studies lack direct evidence identifying its precise RNA substrates and causal signaling pathways in specific immune cell types. In addition, the context-dependent nature of ALKBH5 activity remains unclear, as its functions may vary across tumor types, genetic backgrounds, and microenvironmental conditions such as hypoxia or metabolic stress. Another major gap is cell-type specificity-most work focuses on cancer cells, while the roles of ALKBH5 in dendritic cells, macrophages, NK cells, B cells, and distinct T-cell subsets remain largely unexplored. Moreover, the temporal dynamics of ALKBH5 during tumor progression and treatment are poorly defined, leaving open questions about when and how its modulation affects anti-tumor immunity. The selectivity of ALKBH5 for different RNA substrates, and its functional redundancy or crosstalk with other m^6^A regulators such as METTL3 or FTO, also require further investigation. Translational challenges persist as well: the therapeutic feasibility, safety, and specificity of targeting ALKBH5 have not been established, and its predictive value for responses to immunotherapies like immune checkpoint blockade remains uncertain. Finally, limitations in current experimental models-such as the reliance on murine systems and insufficient validation in human tissues-continue to constrain the generalizability of existing findings. Together, these gaps highlight the need for deeper mechanistic studies, improved models, and more comprehensive clinical validation to fully understand the role of ALKBH5 in tumor immunity.

## 6 Conclusion

In summary, ALKBH5, a critical m^6^A demethylase, exerts complex and context-dependent roles in tumor immune regulation. Through both m^6^A-dependent and independent mechanisms, it influences the fate of immune-related transcripts and modulates key signaling pathways, thereby either promoting immune clearance or driving immune evasion depending on the tumor context. Although current research is still at an early stage, accumulating evidence identifies ALKBH5 as a pivotal regulator of tumor immune responses and immunotherapy outcomes. With deeper mechanistic insights and the development of selective inhibitors, ALKBH5 holds great promise as a novel immunotherapeutic target and predictive biomarker, offering new avenues and strategies to enhance the efficacy and precision of cancer immunotherapy.
